# Antiviral activity of *Cirsium japonicum* var. *spinossimum* extract and its flavonoid cirsimaritin against human coronavirus OC43

**DOI:** 10.71150/jm.2608018

**Published:** 2026-09-30

**Authors:** Chunghyeon Lee, Jang Hoon Kim, Siyun Lee, Seungju Cho, Sumin Kim, Jayhyun Park, Yong-Goo Kim, Junsoo Park

**Affiliations:** 1 Division of Biological Science and Technology, Yonsei University, Wonju 26493, Republic of Korea; 2 Department of Herbal Crop Research, National Institute of Horticultural & Herbal Science, RDA, Eumsung 27709, Republic of Korea; 3 R&D team, Korea Mine Rehabilitation and Mineral Resources Corporation, Wonju 26464, Republic of Korea

**Keywords:** human coronavirus, *Cirsium japonicum* var. *spinossimum* Kitam., cirsimaritin, flavonoid, papain-like protease

## Abstract

Coronaviruses cause a wide range of diseases, ranging from the common cold to severe illnesses such as COVID-19. Although vaccines and therapeutics against COVID-19 have been developed, recent reports have documented the emergence of viral variants with reduced susceptibility to these interventions, suggesting that coronaviruses may continue to circulate persistently, similar to influenza viruses. *Cirsium japonicum* var. *spinossimum* Kitam. (hereafter referred to as *C. japonicum*) has traditionally been used to treat various medical conditions, including inflammatory diseases; however, its antiviral properties remain poorly understood. In this study, we demonstrated that *C. japonicum* extract inhibited human coronavirus replication and alleviated virus-induced cytotoxicity. Analysis of the chemical constituents of the extract identified cirsimaritin, a major flavonoid present in *C. japonicum*, as an active compound with antiviral activity against human coronavirus. The antiviral effects of both *C. japonicum* extract and cirsimaritin were further confirmed using scanning electron microscopy and a three-dimensional spheroid model. Finally, we demonstrated that *C. japonicum* extract and cirsimaritin inhibited coronavirus papain-like protease activity. These findings suggest that *C. japonicum* extract and cirsimaritin have potential as therapeutic candidates for coronavirus-associated diseases.

## Introduction

Recently, the emergence of SARS-CoV-2 has had a profound impact on almost every aspect of human life (Murray et al., 2023; Zhu et al., 2020). However, coronaviruses have circulated in humans long before the COVID-19 pandemic and have been recognized as major causative agents of the common cold. Human coronavirus strains, such as HCoV-OC43, HCoV-229E, HCoV-NL63, and HCoV-HKU1, are widely distributed in the human population and account for approximately 20–30% of common cold cases (Cui et al., 2019; Harrison et al., 2023). As RNA viruses, coronaviruses possess highly mutable genomes, allowing them to evolve rapidly and generate diverse variants or strains (Amoutzias et al., 2022; Markov et al., 2023). Although vaccines and antiviral medicines have been developed against COVID-19, several recent studies have shown that newly emerging SARS-CoV-2 variants can partially evade or resist the protective effects of previously developed vaccines (Carabelli et al., 2023; Dinata et al., 2025; Nooruzzaman et al., 2024; Willett et al., 2022). These findings suggest that coronavirus variants may continue to persist and circulate in the human population, in a manner similar to influenza viruses (Markov et al., 2023; Otto et al., 2024). Therefore, the development of alternative therapeutic strategies against coronaviruses remains necessary, and natural compounds may represent promising candidates for coronavirus treatment.

Several viral enzymes play essential roles in the replication of human coronaviruses and therefore represent attractive targets for antiviral drug development. Among these, the chymotrypsin-like protease (3CLpro, also known as the main protease, Mpro), papain-like protease (PLpro), and RNA-dependent RNA polymerase (RdRp) are considered major therapeutic targets (Wondmkun and Mohammed, 2020; Wu et al., 2020). Following entry into host cells, the coronavirus genomic RNA is translated into two large polyproteins, which are subsequently processed by the viral proteases 3CLpro and PLpro to generate mature functional proteins (Yang and Rao, 2021). Thus, inhibition of these proteases can interfere with the production of functional components and consequently suppress viral replication. Among these proteases, 3CLpro is one of the most extensively validated targets for coronavirus antiviral development. Several clinically available antiviral agents have been developed to inhibit 3CLpro, including nirmatrelvir, the active antiviral component of Paxlovid (nirmatrelvir/ritonavir) (Hammond et al., 2022). In contrast, no PLpro-targeting antiviral drug has yet been widely approved for clinical use. Nevertheless, PLpro is increasingly recognized as an attractive antiviral target because it has dual functions in the coronavirus life cycle (Shin et al., 2020). GRL0617, a well-characterized non-covalent PLpro inhibitor, has consequently been widely used as a reference compound in antiviral studies (Osipiuk et al., 2021). RdRp is another major antiviral target because it catalyzes the synthesis of viral genomic and subgenomic RNAs that are essential for coronavirus replication and gene expression (Malone et al., 2022). Remdesivir and molnupiravir are known to interfere with viral RNA synthesis and suppress viral propagation (Malone et al., 2022).

*Cirsium japonicum* var. *spinossimum* Kitam. (hereafter referred to as *C. japonicum*) is a wild herbaceous plant widely distributed throughout East Asia, including Korea, China, and Japan (Kim et al., 2008). *C. japonicum* has been listed as a medicinal plant in traditional medicine literature and has been used as an anti-hemorrhagic, anti-inflammatory, diuretic, hepatoprotective, and hemostatic agent (Heo, 2013; Li and Luo, 2003; Shin et al., 1998). Recently, various studies have been conducted to investigate the pharmacological activities of *C. japonicum*. First, *C. japonicum* extract has been reported to exhibit anti-inflammatory and antioxidant activities, and cirsimaritin, a flavonoid isolated from *C. japonicum* extract, has also shown anti-inflammatory activity (Kim et al., 2024; Shin et al., 2017). *C. japonicum* extract has also demonstrated potential hepatoprotective effects in both high-fat diet-induced animal models and hepatic cell models (Che et al., 2021; Wan et al., 2014). In addition, treatment with *C. japonicum* extract has shown neuroprotective effects against amyloid beta peptide- and hydrogen peroxide-induced cellular damage (Lee et al., 2018; Pang et al., 2023). Consistent with these pharmacological activities, recent studies have reported that *C. japonicum* extract and cirsimaritin modulate several cell signaling pathways, including NF-κB, AKT, and AMPK signaling (Wan et al., 2014; Yan et al., 2018).

Although many studies have investigated the pharmacological activities of *C. japonicum*, research on its antiviral activity remains limited. Cirsimaritin, a major flavonoid found in *C. japonicum*, has been reported to exhibit antiviral potential against human influenza virus (Yan et al., 2018). In this study, we evaluated the antiviral activity of *C. japonicum* extract against human coronavirus and demonstrated that treatment with *C. japonicum* extract efficiently inhibited coronavirus replication. These findings suggest that *C. japonicum* extract and its major flavonoid cirsimaritin may serve as potential antiviral candidates against human coronavirus.

## Materials and Methods

### Plant materials

The leaves of *C. japonicum* were collected from a farmhouse in Imsil, Jeollabuk-do, Republic of Korea, on July 30, 2022. The species identification was performed by Dr. J. H. Kim. A voucher specimen (CJ220730) was deposited in the herbarium of the Department of Herbal Crop Research, National Institute of Horticultural and Herbal Science.

### Extraction and isolation

The leaves of *C. japonicum* (2.7 kg) were extracted twice with ethanol (36 L) at room temperature for a total of 20 days. The ethanol extract was filtered and concentrated under reduced pressure to obtain a dark brown gum (260 g). The concentrate was suspended in distilled water (2.7 L) and sequentially partitioned with n-hexane, chloroform, and ethyl acetate to yield the n-hexane (93 g), chloroform (42 g), ethyl acetate (70 g), and aqueous fractions (56 g), respectively. The ethyl acetate fraction was further separated by silica gel column chromatography using a chloroform–methanol gradient solvent system (20:0 to 1:1) to yield five fractions (CEE1–CEE5). CEE3 was further separated on a C18 column using a water–methanol gradient solvent system (1:0 to 1:1), yielding compounds 1 (10 mg) and 2 (310 mg). The chloroform fraction was chromatographed by silica gel column chromatography using a chloroform–methanol gradient solvent system (40:0 to 3:1) to yield three fractions (CJC1–CJC3). The CJC2 fraction was further separated by C18 column chromatography using an isocratic solvent system of 60% methanol to yield compound 3 (25 mg).

### HPLC analysis

HPLC analysis was performed on the ethanol extract, its fractions, including n-hexane, chloroform, ethyl acetate, and water fractions, and isolated compounds using an Agilent 1260 HPLC system (Agilent Technologies, USA). The mobile phase consisted of distilled water containing 0.1% formic acid and acetonitrile containing 0.1% formic acid. Gradient elution was performed as follows: 5–55% acetonitrile for 0–20 min, 55–100% acetonitrile for 20.1–27 min, 100% acetonitrile for 27.1–30 min, and 5% acetonitrile for 30.1–35 min. The samples were analyzed using an Eclipse Plus C18 column (5 µm, 4.6 × 250 mm). The flow rate was 1.0 ml/min, and the injection volume was 10 µl. The sample concentrations were 5 mg/ml for the extract and fractions and 0.25 mg/ml for the isolated compounds. Detection was performed at 254 nm. The calibration curve for quantification was constructed using standard concentrations ranging from 3.9 to 250 µg/ml and the corresponding HPLC peak areas.

### Infection of coronavirus

HCoV-OC43 was purchased from American Type Culture Collection (ATCC, USA), and rhabdomyosarcoma (RD) cells were purchased from the Korean Cell Line Bank. To maintain RD cells, the cells were cultured in DMEM medium (Welgene, Republic of Korea) supplemented with 10% fetal bovine serum (FBS; Thermo Fisher Scientific, USA) and 1% penicillin–streptomycin solution (Welgene). To establish the 3D spheroid culture model, 2 × 10^4^ RD cells were seeded into each well of a 96-well Ultra low attachment plate (Corning Life Sciences, USA) and subsequently centrifuged at 200 × g. The cells were then incubated at 37°C for 2 days to allow spheroid formation.

Coronavirus infection was performed using a previously established protocol (Jang et al., 2021). Briefly, RD cells were inoculated with virus-containing medium at an MOI of 0.01 using the indicated dilutions. After viral infection, the cells were incubated in MEM (Welgene) supplemented with 2% FBS and 1% penicillin–streptomycin. Cell viability was evaluated by MTT assay according to a previously described protocol (Jang et al., 2018).

### Plaque formation assay

Plaque formation assay was performed to measure the amount of infectious coronavirus titer. 8 × 10⁴ cells of RD were seeded in each well of 12-well plates and then infected with the indicated dilutions of coronavirus. After 1 h of infection, the cells were overlaid with 2× MEM (Welgene) containing 0.3% Agarose. The plates were incubated at 33°C for 4 days to allow plaque formation. Cells were fixed with 4% paraformaldehyde and visualized using 0.25% crystal violet solution.

### Western blot

Coronavirus protein expression was analyzed by Western blotting with an anti-HCoV-OC43 antibody. Cell lysates and conditioned media were harvested independently and prepared using cell lysis buffer containing 150 mM NaCl, 50 mM HEPES (pH 7.5), and 1% NP-40, supplemented with a protease inhibitor cocktail (Roche, Germany). Protein concentrations were determined using the Bradford assay, and equivalent amounts of protein samples were separated by SDS-PAGE and subsequently transferred to PVDF membranes (Cytiva, USA). The membranes were blocked in 3% skim milk prepared in TBS-T buffer (TBS with 0.1% Tween-20) and incubated with an anti-HCoV-OC43 primary antibody (Sigma-Aldrich, USA). Protein bands were detected using the ChemiDoc Imaging System (Bio-Rad, USA).

### Quantitative RT-PCR

Quantitative RT-PCR analysis was conducted to determine coronavirus RNA levels in both cell lysates and conditioned media. Cells and culture supernatants were harvested separately, and total RNA was isolated using the PURETM Total RNA Extraction Kit (Infusion Tech, Republic of Korea) following the manufacturer’s protocol. Equivalent quantities of RNA were then reverse-transcribed into cDNA using the M-MLV cDNA Synthesis Kit (Enzynomics, Republic of Korea). Real-time PCR amplification was performed using 2X Real-Time PCR Master Mix including SYBR Green (BioFact, Republic of Korea) on the QuantStudio 3 Real-Time PCR System, and RPL4 gene expression was used for normalization. An equal amount of RNA isolated from uninfected cells was added to each conditioned medium sample as carrier RNA to enhance viral RNA recovery and provide a consistent RNA background for subsequent analysis. Primer sets targeting viral and cellular genes were adopted from a previously published study (Jang et al., 2021).

### Scanning electron microscopy (SEM)

For SEM analysis, RD cells were seeded onto sterilized 9-mm coverslips and infected with HCoV-OC43 for 72 h. Following infection, cells were fixed in 2.5% glutaraldehyde for 1 h and sequentially dehydrated using increasing concentrations of ethanol (20%, 40%, 60%, 80%, 90%, and 100%). The samples were subsequently dried in a vacuum desiccator for 30 min. After platinum coating, cellular morphology was examined using a SUPRA 40 scanning electron microscope manufactured by Carl Zeiss (Germany).

### Immunofluorescence assay

Immunofluorescence assay was conducted to visualize the reduction of coronavirus in the 3D spheroid model. Infected spheroids were fixed using 4% paraformaldehyde and permeabilized using permeabilization buffer containing 0.5% Triton X-100 (Sigma-Aldrich) in PBS. The spheroids were then blocked with PBS containing 5% FBS, 2% BSA, and 0.1% Triton X-100. Coronaviruses were labeled with HCoV-OC43 antibody (Merck, Germany) and Alexa488-conjugated second antibody (Invitrogen, USA). Images were acquired using a Zeiss Axio Imager M2 at the Neuroscience Translational Research Solution Center (Republic of Korea).

### PLpro assay

The pBT7-N-His-PLpro plasmid encoding full-length SARS-CoV-2 PLpro was custom-synthesized by Bioneer (Republic of Korea) and transformed into *Escherichia coli* BL21 (DE3) cells (Enzynomics). Protein expression was induced with 1 mM isopropyl β-D-1-thiogalactopyranoside (IPTG), and the cells were incubated overnight at 18°C. The cells were then harvested and lysed, and His-tagged PLpro was purified from the soluble fraction using Ni-NTA resin (Thermo Fisher Scientific). The resin was washed with buffer containing 25 mM imidazole, and PLpro was sequentially eluted with buffers containing 250 and 500 mM imidazole. The purified protein was dialyzed against 1 L of 1× phosphate-buffered saline (PBS) containing 10% (v/v) glycerol and 0.01% (v/v) β-mercaptoethanol. The dialyzed protein was aliquoted and stored at −80°C until use.

PLpro activity was measured using a fluorescence resonance energy transfer (FRET)-based protease assay. The fluorogenic peptide Dabcyl-FTLRGG/APTKV-Edans, dissolved in dimethyl sulfoxide (DMSO), was used as the PLpro substrate. Purified PLpro and the FRET substrate were used at final concentrations of 0.5 and 20 µM, respectively, in 20 mM HEPES buffer (pH 7.5), with a total reaction volume of 50 µl. The reactions were carried out in black 96-well plates (SPL Life Sciences, Republic of Korea) at 37°C for 60 min. Fluorescence intensity was measured at excitation and emission wavelengths of 360 and 460 nm, respectively, using an Infinite M Plex multimode microplate reader (Tecan, Switzerland). For the inhibition assay, purified PLpro was preincubated with the indicated concentrations of the test materials for 1 h before the addition of the FRET substrate. The final DMSO concentration was kept constant in all reaction mixtures. Reactions containing PLpro and the substrate without a test compound served as enzyme controls, whereas reactions containing the substrate without PLpro were used to determine background fluorescence. PLpro activity was calculated from the increase in fluorescence intensity after subtraction of the substrate-only background. Relative PLpro activity was expressed as a percentage of the activity measured in the compound-free PLpro control. GRL0617 (Sigma-Aldrich) was used as a positive control.

### Statistical analysis

The results of western blotting, quantitative RT-PCR, MTT assay and PL-pro assay were statistically evaluated by one-way ANOVA followed by Dunnett’s multiple comparison test using GraphPad Prism software, version 8.0.2 (GraphPad Software, USA). Differences were considered statistically significant at *p* < 0.05. IC_50_ values were calculated by nonlinear regression analysis using GraphPad Prism.

## Results

### *C. japonicum* extract inhibits the expression of coronavirus proteins

We screened plant extracts for antiviral activity against human coronavirus, and we used Human coronavirus OC-43 strain, which belongs to the genus Betacoronavirus. After screening, we found that *C. japonicum* extract showed the antiviral activity, and we prepared various fractions from *C. japonicum* extract to identify the antiviral compounds (Fig. 1A). Fractionation of the *C. japonicum* ethanol extract was subsequently performed to identify the fraction containing antiviral activity (Fig. 1A). Among the tested fractions, the chloroform and ethanol fractions markedly reduced HCoV-OC43 protein expression (Figs. 1B and S1). In particular, viral protein expression in conditioned medium was barely detectable following treatment with 20 µg/ml of either fraction. These results indicate that chloroform fraction as well as ethanol fraction contain the antiviral materials.

### *C. japonicum* extract inhibits coronavirus replication

Because the chloroform fraction of *C. japonicum* extract (hereafter referred to as CJE) was shown to inhibit the coronavirus protein expression, we analyzed the antiviral effects with various concentrations of CJE. When the cells were infected and treated with CJE, we analyzed the level of coronavirus proteins in the cell lysates and conditioned media. We found that CJE treatment decreased the expression of coronavirus proteins in the cell lysates, and moreover, CJE treatment reduced the coronavirus protein expression in the conditioned medium more efficiently (Figs. 2A and S2). The half maximal inhibitory concentration (IC_50_) was calculated as 8.33 µg/ml in the cell lysates and 2.5 µg/ml in the conditioned medium (Figs. 2A and S2). Next, we analyzed the level of human coronavirus RNA by using quantitative RT-PCR. We next quantified viral RNA levels by qRT-PCR using primer sets targeting the RNA-dependent RNA polymerase (RdRp), membrane protein (M), and nucleocapsid protein (N) to measure the RNA levels. CJE treatment reduces the level of RdRp, M and N in a dose-dependent manner in cell lysates and conditioned media (Fig. 2B). Similar to the protein level, the reduction in the conditioned media is more dramatic, and IC_50_ was 5.09–7.12 µg/ml in the cell lysates and 2.34–2.81 µg/ml in the conditioned medium (Fig. 2B). Together, the reductions in intracellular and extracellular viral RNA and protein levels suggest that CJE suppresses HCoV-OC43 replication.

### CJE ameliorates coronavirus-induced cytotoxicity

Coronavirus infection shows the cytopathic effect to the infected cells, and the antiviral compounds often ameliorate the coronavirus-induced cytotoxicity. Because CJE treatment inhibits the coronavirus replication, we examined the effect of CJE on the coronavirus-infected cells. First, we examined whether CJE treatment affects the cell viability, and CJE treatment did not decrease the cell viability up to 20 µg/ml (Fig. 3A). Next, we examined whether CJE treatment ameliorates the coronavirus-induced cytotoxicity. When the cells were infected with coronavirus, the viability was reduced up to 40% of control cells, CJE reduced the coronavirus-induced cytotoxicity in a dose-dependent manner (Fig. 3B). We also observed the decrease of cytopathic effects by the light microscopy, and the floating and dead cells were decreased by CJE treatment (Fig. 3C). Finally, we evaluated the effect of CJE on infectious virus production using a plaque assay. While coronavirus infection results in the formation of a large number of plaques, CJE treatment decreased the number of plaques formation in a dose-dependent manner (Fig. 3D). These results indicate that CJE treatment ameliorates coronavirus-induced cytopathic effects.

### Component analysis of *C. japonicum* extract

Because *C. japonicum* extract showed the antiviral effects, we wanted to identify the active compounds in the *C. japonicum* extract. We analyzed the components of *C. japonicum* extract by HPLC methods, and ethanol fraction, and n-hexane, chloroform, ethyl acetate, and water fractions were used for analysis (Fig. 4A). We isolated three components from chloroform and ethyl acetate fractions of the *C. japonicum* extracts, respectively, they are hispidulin-4'-*O*-glucoside (1), cirsimarin (2), and cirsimaritin (3) (Fig. 4B). Single compounds 1, 2, and 3 exhibited the retention time of HPLC signals at 13.4 min, 15.3 min, and 22.1 min, respectively. It was confirmed through HPLC signals that the compound 3 was present in the highest amount in the chloroform fraction (Fig. 4). The relative amounts of each compound in ethanol extract were calculated (Table 1). We analyzed the effects of each compound on the expression of coronavirus proteins. The inhibitory effect of hispidulin-4'-*O*-glucoside (1) and cirsimarin (2) was marginal on the expression of coronavirus, however cirsimaritin (3) treatment decreased the expression of coronavirus significantly (Figs. 4C and S3). These results suggest that cirsimaritin isolated from *C. japonicum* extract shows the inhibitory activity against coronavirus.

### Cirsimaritin treatment inhibits the coronavirus replication

Because cirsimaritin isolated from *C. japonicum* extract inhibits the expression of coronavirus proteins, we examined the antiviral effects of cirsimaritin. First, we determined the effective concentration of cirsimaritin. When the cells were treated with cirsimaritin, the cell viability was decreased at 30 µM and 40 µM, however the cell viability was not significantly decreased up to 20 µM (Fig. 5A). Next, we examined whether cirsimaritin ameliorates coronavirus-induced cytotoxicity, and cirsimaritin treatment significantly decreased the coronavirus-induced cytotoxicity (Fig. 5B). We also examined the effect of cirsimaritin treatment upon plaque formation. Coronavirus infection results in the plaque formation, and cirsimaritin treatment significantly reduces the formation of coronavirus induced plaque (Fig. 5C). These results indicate that cirsimaritin treatment ameliorates coronavirus induced cytotoxicity like CJE.

Next, we measured the IC_50_ of cirsimaritin for coronavirus inhibition by examining the coronavirus protein expression. Cirsimaritin treatment inhibited the expression of coronavirus proteins in the cell lysates and conditioned media in a dose dependent manner (Figs. 5D and S4). IC_50_ values of cirsimaritin in the cell lysates and conditioned media are 13.06 µM and 5.03 µM respectively (Fig. 5E). These results indicate that cirsimaritin isolated from CJE inhibits the coronavirus replication.

### CJE and cirsimaritin inhibit the coronavirus production

We demonstrated that both CJE and cirsimaritin, isolated from CJE, inhibit the coronavirus replication in the cells. Next, we attempted to observe the effect of CJE directly in the cells using scanning electron microscopy. Cells were infected with coronavirus, treated with CJE and cirsimaritin and visualized to observe the coronavirus production. Coronavirus infection resulted in production of many virions on the cell membrane, and either CJE or cirsimaritin treatment decreased the number of coronavirus particles in the membrane (Fig. 6). These results indicate that CJE and cirsimaritin inhibits the coronavirus production in the infected cells.

### CJE and cirsimaritin inhibits the coronavirus replication in 3D spheroid culture model

Compared with conventional 2D monolayer culture, 3D culture models are considered more biologically relevant because they better mimic the in vivo cellular microenvironment (Edmondson et al., 2014; Kapałczyńska et al., 2018). Therefore, we attempted to examine the antiviral effect of CJE and cirsimaritin against coronavirus using a 3D spheroid culture model. 3D spheroids were formed and infected with human coronavirus and incubated with various concentrations of CJE. 3D spheroids were stained with coronavirus antibody along with DAPI for the nucleus. Coronavirus infection was observed throughout the 3D spheroid and CJE treatment reduced the coronaviral protein expression in the 3D spheroid (Fig. 7A). Coronavirus infection was detected in the limited part of spheroids upon CJE treatment (Fig. 7A). Next, we evaluated the coronavirus RNA expression in the spheroids. Surprisingly, CJE treatment decreased the coronavirus RNA level dramatically, and IC_50_ was 1.15–1.99 µg/ml (Fig. 7B).

Next, we examined the antiviral effect of cirsimaritin using 3D spheroid model. Similar to CJE, cirsimaritin treatment reduces the coronavirus infection, and the number of coronavirus positive cells was decreased (Fig. 7C). We also evaluated the level of coronavirus RNA in the spheroids, and IC_50_ of cirsimaritin was 7.40–11.94 µM (Fig. 7D). These results indicate that cirsimaritin inhibits the coronavirus infection in 3D spheroid culture.

### Cirsimaritin inhibits the papain-like protease

Because both CJE and cirsimaritin inhibited coronavirus replication, we next investigated their potential molecular targets. Papain-like protease (PLpro) and chymotrypsin-like protease (3CLpro) are two major coronavirus proteases and important targets for antiviral drug development. We initially examined the effects of CJE and cirsimaritin on 3CLpro activity; however, neither treatment produced a significant inhibitory effect (data not shown). We therefore evaluated their effects on PLpro activity and found that both CJE and cirsimaritin significantly inhibited PLpro activity (Fig. 8). GRL0617, a well-characterized PLpro inhibitor, was used as a positive control. These results suggest that the antiviral effects of CJE and cirsimaritin may be mediated, at least in part, through the inhibition of coronavirus PLpro.

## Discussion

In this study, we demonstrated the antiviral effects of *C. japonicum* extract, and cirsimaritin, a flavonoid isolated from *C. japonicum* extract. Initially, we used RD cells for infection and the treatment of *C. japonicum* extract inhibits the expression level of coronavirus proteins and the level of coronavirus RNA in the cells and conditioned medium. We wanted to isolate the active antiviral components in *C. japonicum* extract, and showed that cirsimaritin, one component of *C. japonicum* inhibits the coronavirus replication similar to *C. japonicum* extract. We used scanning electron microscopy (SEM) to visualize the antiviral effects, and the SEM image revealed the clear reduction of produced coronavirus particles upon the CJE treatment.

We used 3D cell culture model to mimic the real virus infection in animal tissues. Animal experiments were commonly used to examine the antiviral in vivo, however nowadays 3D spheroid models were often used to replace to examine the biological relevance of cell culture systems (Ebisudani et al., 2021; Urzì et al., 2023). 3D spheroids were formed using low attachment bottom plates, the infection was visualized by immunofluorescence. Because the spheroids were small, we were not able to examine the expression of coronavirus proteins by Western blot. Therefore, we evaluate the level of coronavirus RNA by qRT-PCR. When we compared the IC_50_ between 2D culture and 3D spheroid model, the IC_50_ in 3D spheroid model (1.15–1.99 µg/ml) was much lower than 2D culture (5.09–7.12 µg/ml). The lower IC_50_ observed in the 3D spheroid model indicates that the antiviral activity of CJE may be influenced by the cellular microenvironment and culture architecture. The enhanced antiviral effect observed in 3D spheroids may reflect differences in virus spread, cell-cell interactions, drug accessibility, or cellular responses compared with conventional monolayer cultures. Further studies specifically designed to distinguish cell-free from cell-to-cell viral transmission will be required to determine whether CJE affects these modes of viral spread.

When comparing the antiviral activity of *C. japonicum* extract and cirsimaritin, the viral protein expression results showed that cirsimaritin exerted an effect comparable to that of the extract. Although direct comparison between a complex extract and a purified compound should be interpreted cautiously, the IC₅₀ of *C. japonicum* extract was 2.5 µg/ml, whereas the IC₅₀ of cirsimaritin was 1.58 µg/ml, equivalent to 5.03 µM. These results support the notion that cirsimaritin is an active antiviral component of *C. japonicum* extract. However, it is also possible that CJE contains additional minor antiviral components that inhibit coronavirus replication.

After demonstrating that both *C. japonicum* extract and cirsimaritin exert antiviral activity against human coronavirus, we sought to investigate the molecular mechanisms underlying their inhibitory effects on viral replication. Coronavirus proteases, including the 3C-like protease (3CLpro) and papain-like protease (PLpro), are well-established targets for the development of coronavirus antivirals. We initially examined whether *C. japonicum* extract and cirsimaritin inhibited 3CLpro activity; however, neither treatment produced a significant inhibitory effect. We subsequently evaluated their effects on PLpro activity and found that both *C. japonicum* extract and cirsimaritin significantly inhibited PLpro. GRL0617, a well-known PLpro inhibitor, was used as a positive control and exhibited stronger inhibitory activity than either *C. japonicum* extract or cirsimaritin. These findings suggest that the inhibition of PLpro may contribute, at least in part, to the suppression of coronavirus replication by *C. japonicum* extract and cirsimaritin. Nevertheless, additional molecular targets or mechanisms may also be involved in their antiviral effects.

*Cirsium japonicum* var. *spinossimum* Kitam. (*C. japonicum*) has been described as a medicinal plant in traditional medical literature. In this study, we showed that treatment with *C. japonicum* extract inhibited the replication of human coronavirus. We also analyzed the chemical constituents of *C. japonicum* extract and demonstrated that cirsimaritin is an active antiviral compound present in the extract. In addition, we confirmed the antiviral effects of *C. japonicum* extract and cirsimaritin using a 3D spheroid model, which provides a more biologically relevant system for evaluating antiviral activity. These results suggest that *C. japonicum* extract and cirsimaritin may serve as potential sources of antiviral agents against human coronavirus.

## Figures and Tables

**Fig. 1.**
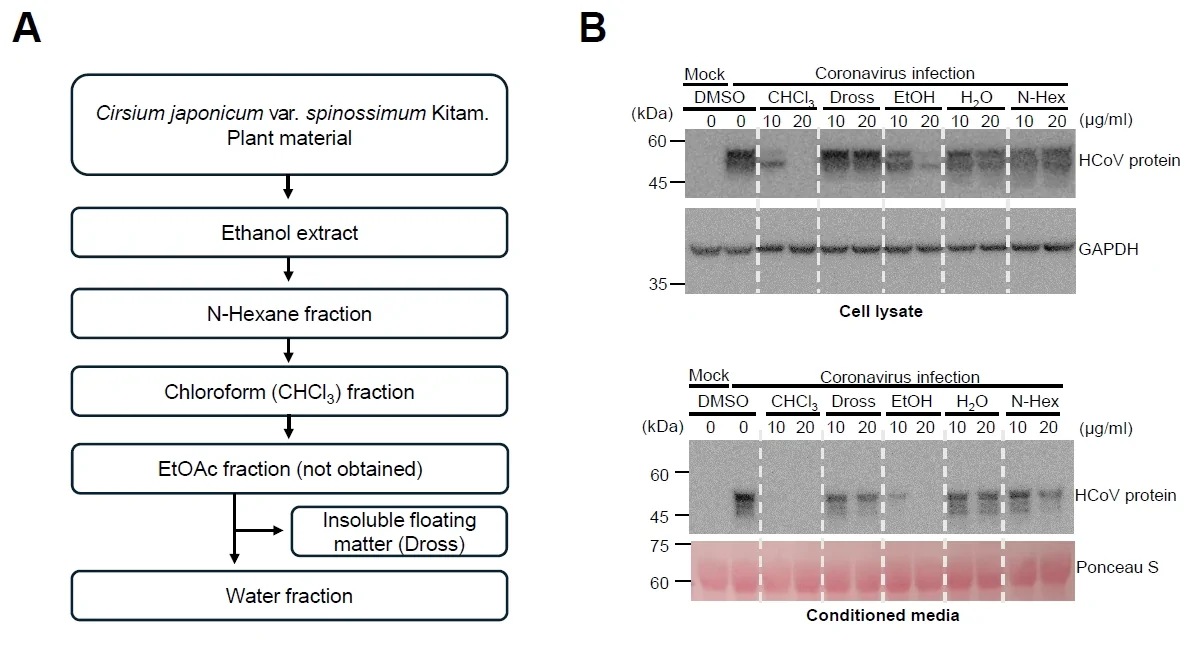
*C. japonicum* extract reduces the expression of human coronavirus proteins. (A) Fractionation scheme of *C. japonicum* extract. (B) The chloroform fraction of *C. japonicum* extract (CJE) reduced coronavirus protein expression. RD cells were infected with HCoV-OC43 and simultaneously treated with *C. japonicum* leaf extract or its fractions for 72 h. Cells and conditioned media were then collected, and viral protein levels were analyzed by Western blotting. GAPDH and Ponceau S staining were used as loading controls.

**Fig. 2.**
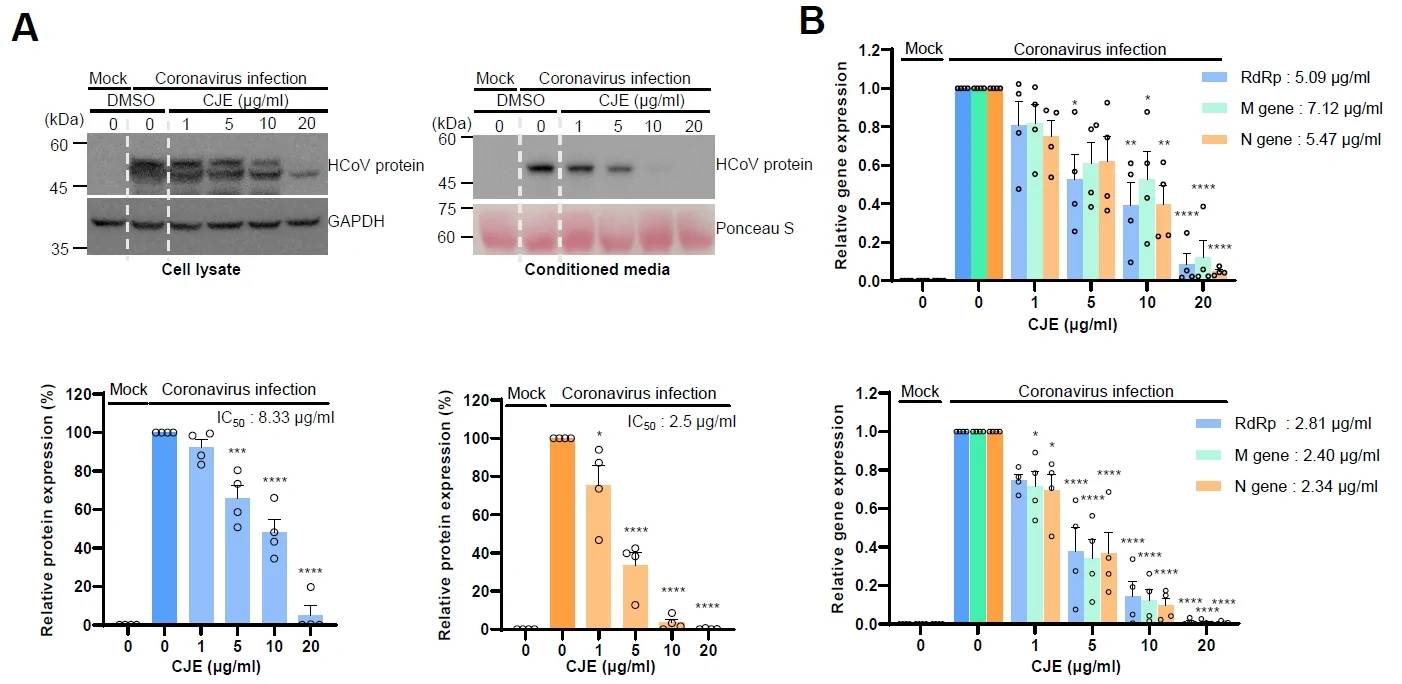
CJE inhibits coronavirus replication and protein synthesis in a dose-dependent manner. (A) CJE inhibits coronavirus protein expression. RD cells were treated with coronavirus and CJE. Viral protein expression was analyzed by Western blotting (upper panel). Cell lysates and conditioned media were collected, and the graphs show the relative levels of coronavirus protein expression (lower panel). Data are presented as the Mean ± SEM from four independent experiments (N = 4). (B) CJE reduces viral RNA levels in a dose-dependent manner. Viral RNA levels in infected cells (upper panel) and conditioned media (lower panel) were measured by qRT-PCR after treatment with increasing concentrations of CJE. Relative RNA levels were normalized to RPL4 and are presented as fold change relative to the infected control. Error bars represent SEM (N = 4). Statistical significance was determined using one-way ANOVA followed by Dunnett’s multiple-comparisons test. Compared with the infected control, *P < 0.05, **P < 0.01, ***P < 0.001, and ****P < 0.0001.

**Fig. 3.**
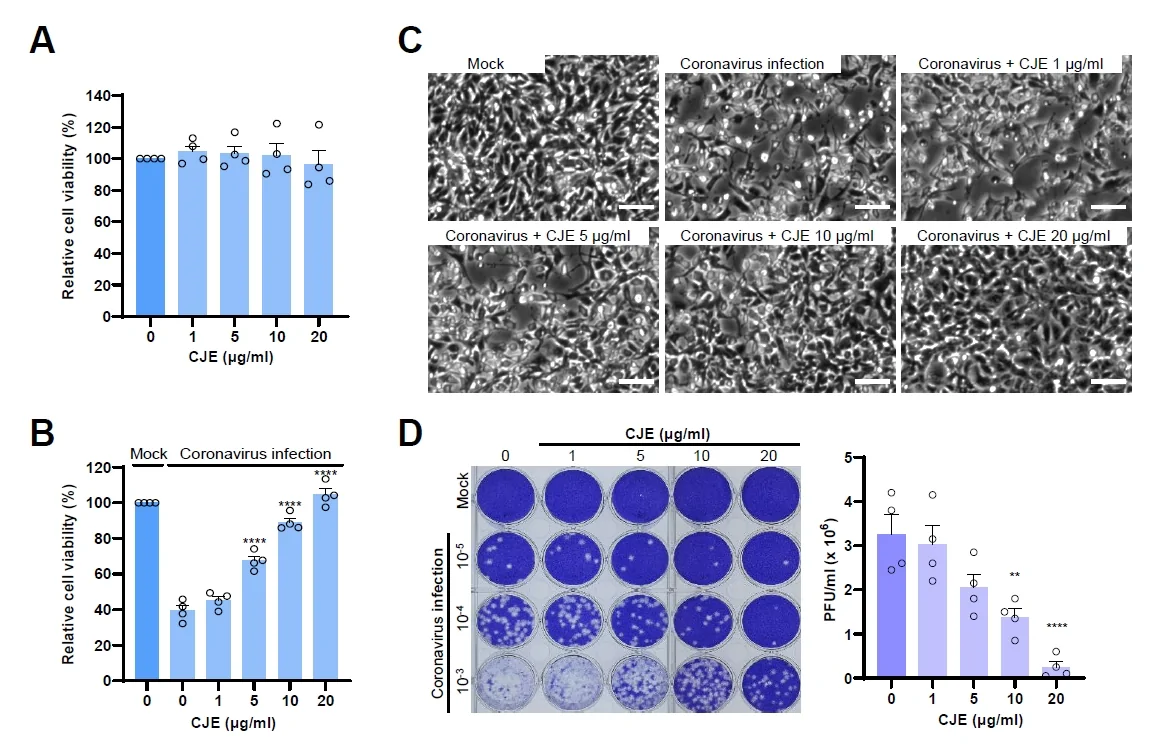
CJE exhibits a protective effect against coronavirus-induced cytotoxicity. (A) RD cells were treated with the indicated concentrations of CJE for 24 h, and cell viability was measured using the MTT assay. (B) RD cells were simultaneously treated with CJE and infected with coronavirus, and cell viability was assessed by the MTT assay. (C) Representative microscopic images of coronavirus-infected RD cells treated with CJE. CJE treatment improved cell viability under coronavirus-infected conditions. Scale bars = 10 μm. (D) RD cells were infected with serially diluted coronavirus and simultaneously treated with CJE for 96 h. Plaque formation was visualized by crystal violet staining, and the number of plaques was counted. The viral titers were calculated and expressed as plaque-forming units per milliliter (PFU/ml). Error bars represent SEM (N = 4). Statistical significance was determined using one-way ANOVA followed by Dunnett’s multiple-comparisons test. Compared with the infected control, *P < 0.05, **P < 0.01, ***P < 0.001, and ****P < 0.0001.

**Fig. 4.**
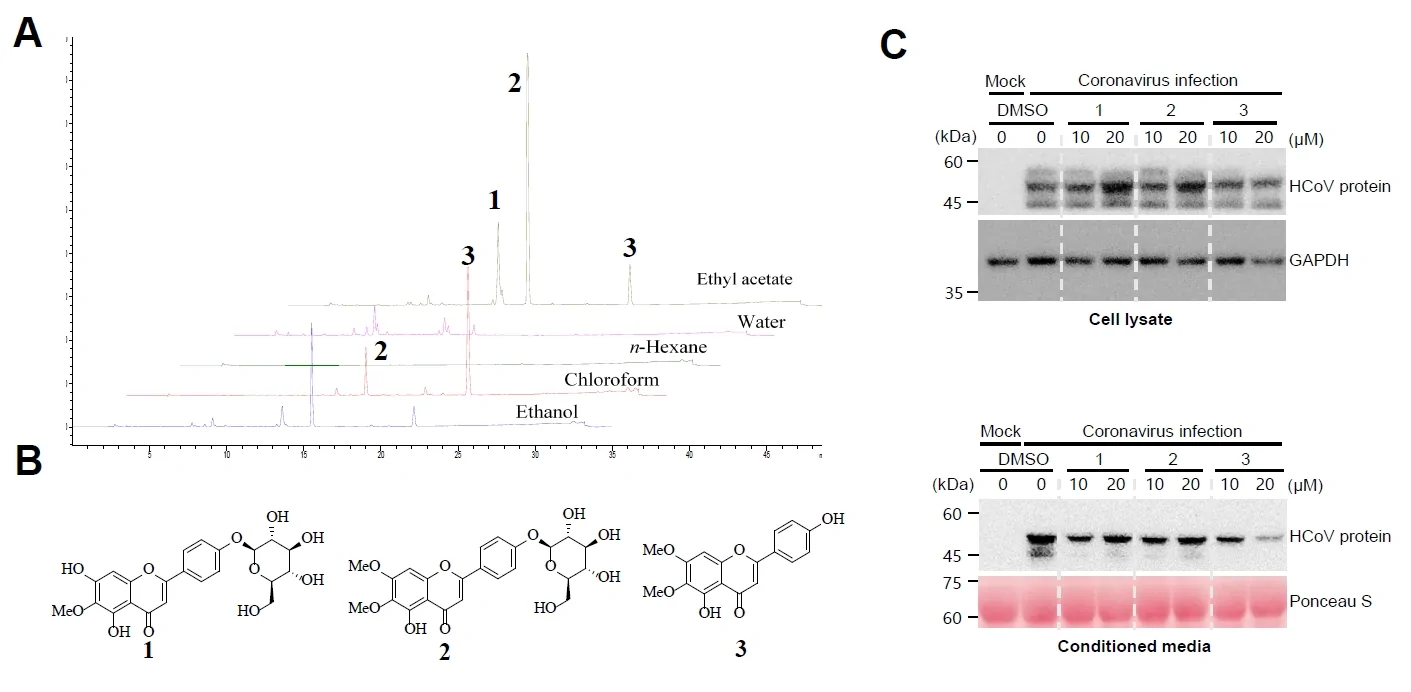
Isolation of active antiviral compounds in *C. japonicum* extract. (A) HPLC chromatograms of the ethanol extract, and n-hexane, chloroform, ethyl acetate and water fractions prepared from the leaves of *C. japonicum* (B) Chemical structures of compounds 1–3 isolated from the leaves of *C. japonicum*, 1, hispidulin-4′-*O*-glucoside; 2, cirsimarin; and 3, cirsimaritin. (C) Cirsimaritin markedly reduced viral protein expression. Western blot analysis was performed to evaluate the antiviral effects of individual compounds from CJE. RD cells were infected with coronavirus and treated with the indicated concentrations of the compounds for 72 h.

**Fig. 5.**
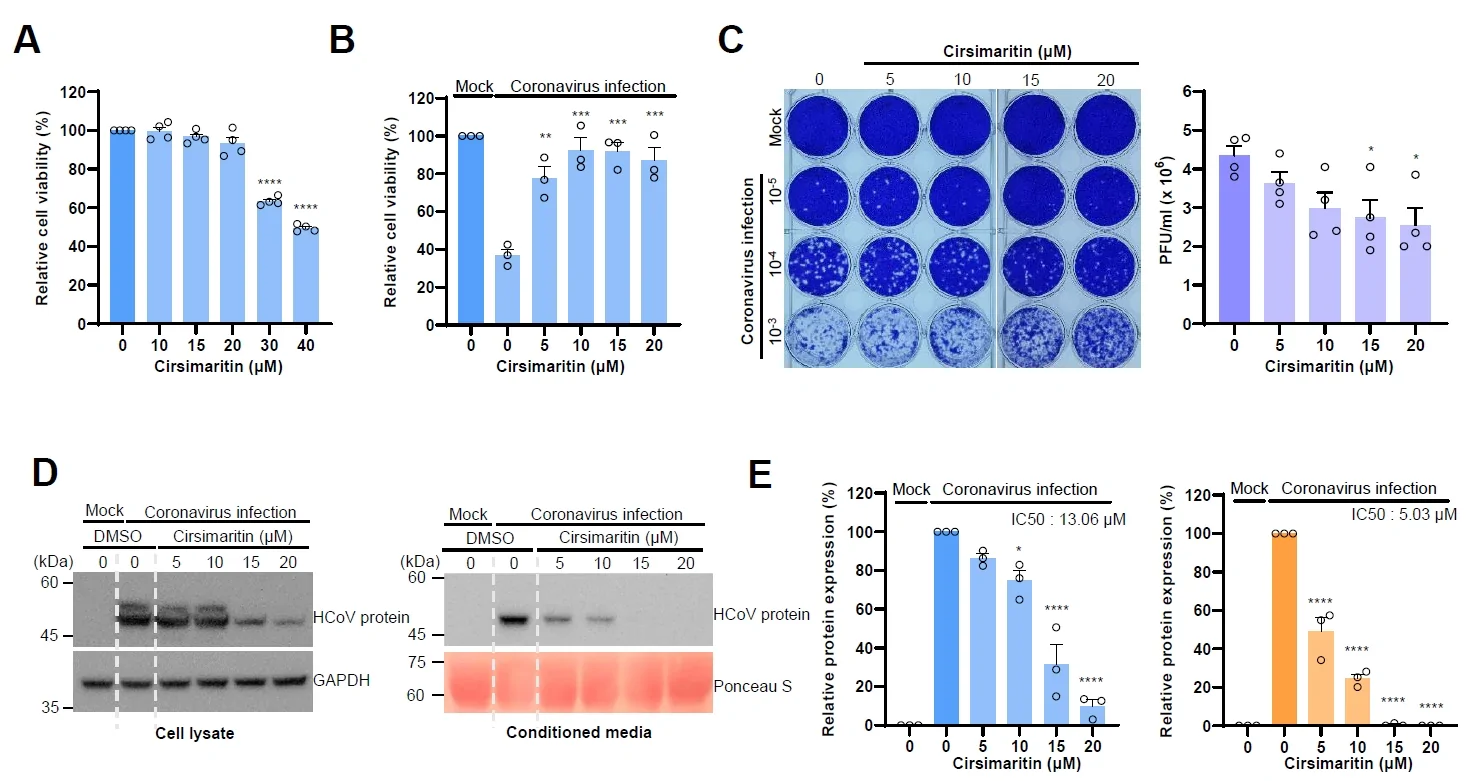
Cirsimaritin treatment inhibits coronavirus replication. (A) RD cells were treated with the indicated concentrations of cirsimaritin for 24 h, and cell viability was measured using the MTT assay. (B) The viability of coronavirus-infected RD cells was assessed by the MTT assay following treatment with cirsimaritin. (C) RD cells were infected with serially diluted coronavirus and simultaneously treated with increasing concentrations of cirsimaritin. Plaque formation was quantified, and the calculated viral titers were shown in the graph (N = 4). (D) Viral protein expression in cell lysates and conditioned media was analyzed by Western blotting following cirsimaritin treatment. (E) The graphs show the relative levels of coronavirus protein expression in cell lysate (left) and conditioned media (right). Error bars represent SEM (N = 4). Statistical significance was determined using one-way ANOVA followed by Dunnett’s multiple-comparisons test. Compared with the infected control, *P < 0.05, **P < 0.01, ***P < 0.001, and ****P < 0.0001.

**Fig. 6.**
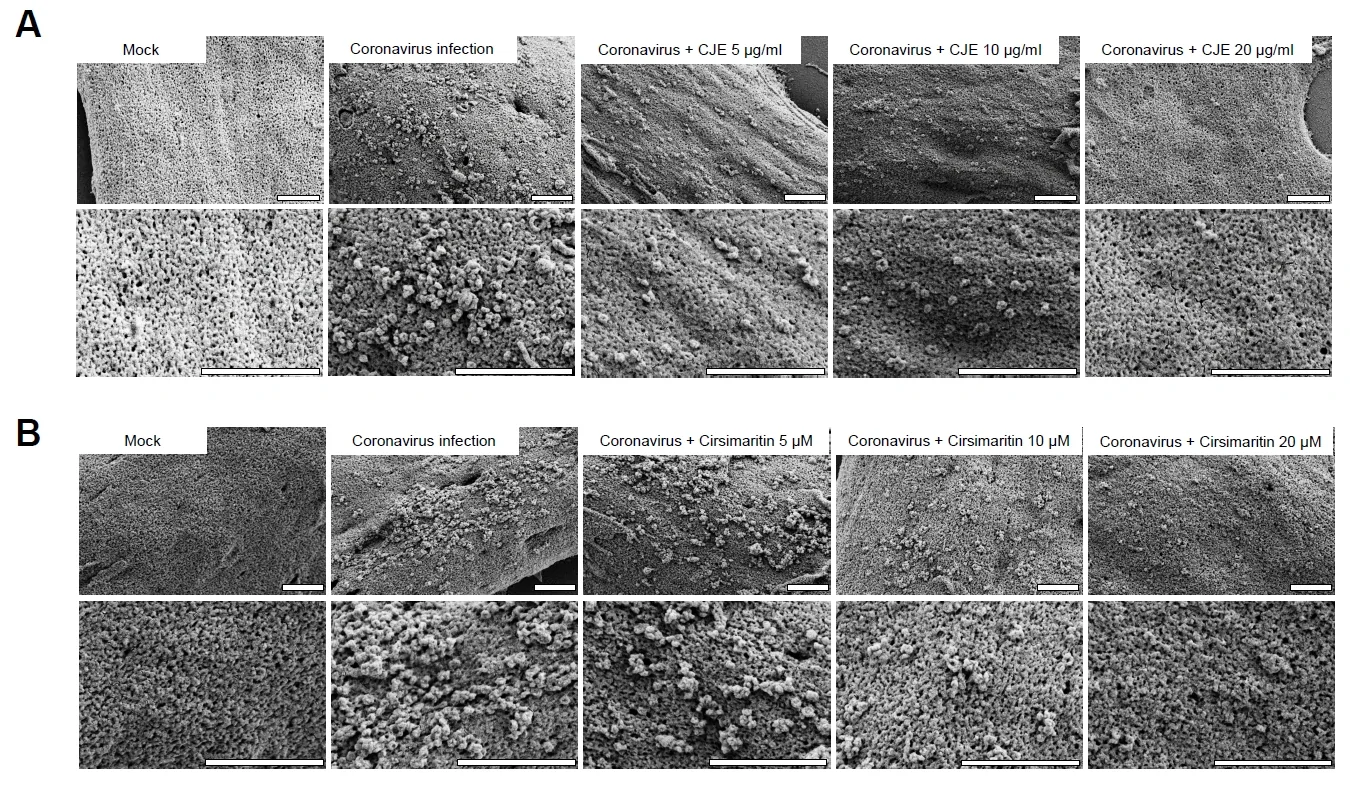
Treatment with CJE and cirsimaritin decreases the number of coronavirus particles in the virus-infected cell surface. Scanning electron microscopy (SEM) images of coronavirus-infected cells treated with (A) CJE and (B) cirsimaritin. The number of coronavirus particles on the cell surface was reduced in both treatment groups. Scale bars = 1 µm.

**Fig. 7.**
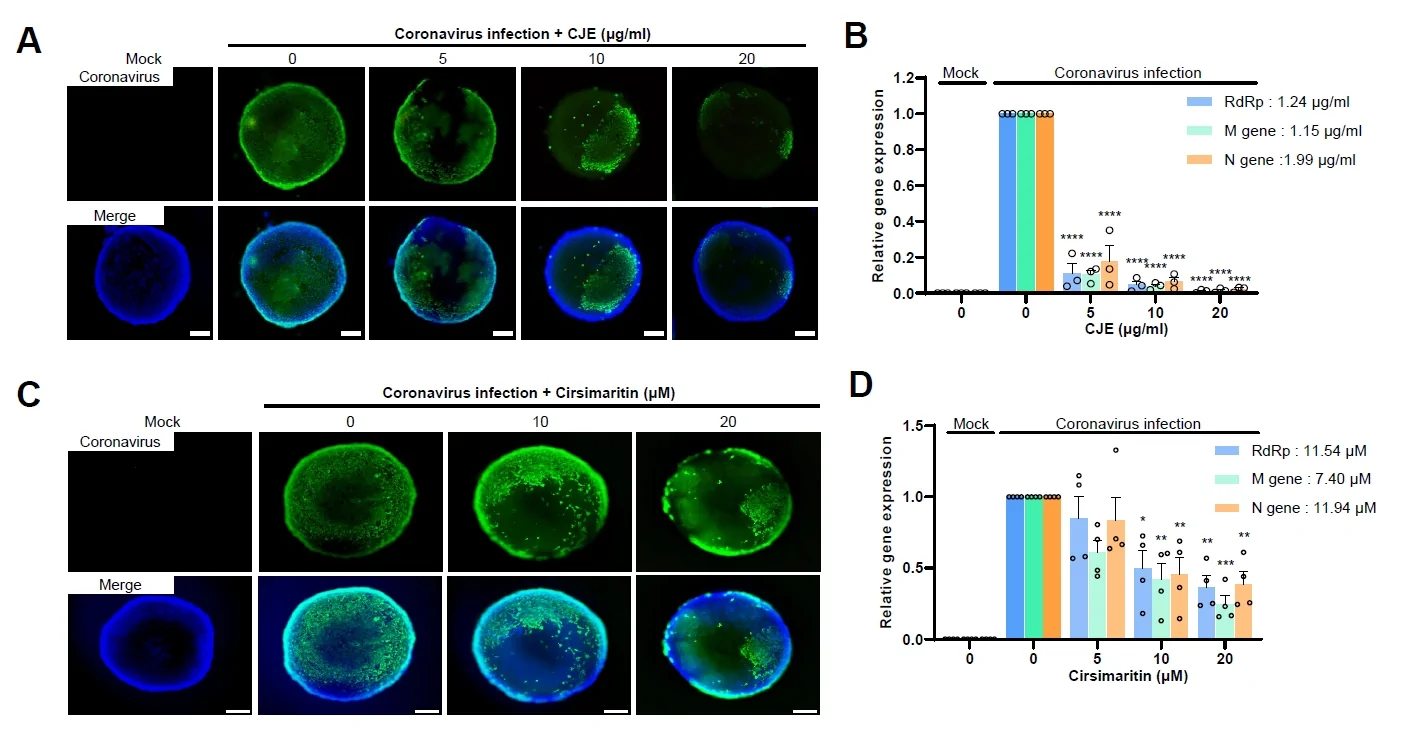
CJE and cirsimaritin inhibit coronavirus replication in a 3D spheroid culture model. (A) Immunofluorescence images showing the inhibitory effect of CJE on coronavirus replication in 3D spheroids. Scale bars = 200 µm. (B) Coronavirus RNA levels in 3D spheroids were quantified following infection and treatment with CJE. The IC_50_ value of CJE was determined from the dose–response curve. Data were normalized to RPL4 and are presented as Mean ± SEM (N = 3). (C) Immunofluorescence images showing the inhibitory effect of cirsimaritin on coronavirus replication in 3D spheroids. (D) Coronavirus RNA levels following cirsimaritin treatment were quantified and are shown in the graph. Data were normalized to RPL4 and are presented as Mean ± SEM (N = 4). Statistical significance was determined using one-way ANOVA followed by Dunnett’s multiple-comparisons test. Compared with the infected control, *P < 0.05, **P < 0.01, ***P < 0.001, and ****P < 0.0001.

**Fig. 8.**
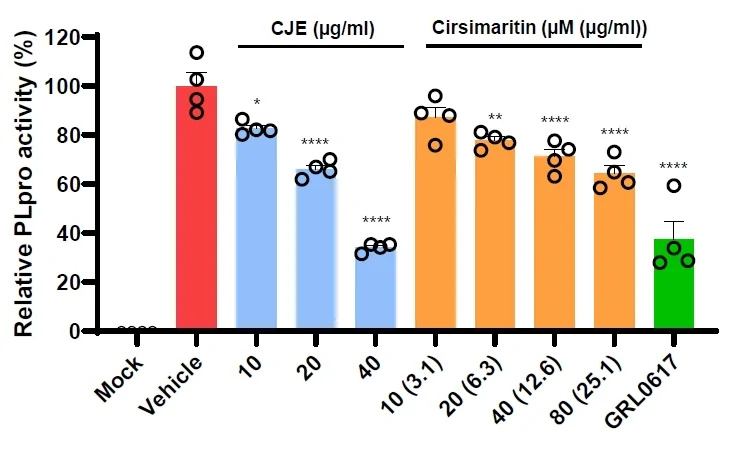
CJE and cirsimaritin inhibit coronavirus PLpro activity. The indicated concentrations of CJE and cirsimaritin were incubated with PLpro, and protease activity was subsequently measured. GRL0617 (10 µM) was used as a positive control. PLpro activity was evaluated in quadruplicate, and the data are presented as the Mean and the standard deviation (SD). Statistical significance was determined using one-way analysis of variance (ANOVA), followed by Dunnett’s multiple-comparison test against the vehicle-treated control (0). *P < 0.05, **P < 0.01, and ****P < 0.0001.

**Table 1.** Contents compounds 1–3 in ethanol extract of *C. japonicum* leaves

Compound	Contents (mg/g, %)
1	12.3 (1.2)
2	43.67 (4.37)
3	5.38 (0.54)

## Supplementary Information

Fig. S1. Uncropped original Western blot images for Fig. 1B.

Fig. S2. Uncropped original Western blot images for Fig. 2A.

Fig. S3. Uncropped original Western blot images for Fig. 4C.

Fig. S4. Uncropped original Western blot images for Fig. 5D.
