# Supplementary material for: Antiviral activity of Cirsium japonicum var. spinossimum extract and its flavonoid cirsimaritin against human coronavirus OC43
Source: J Microbiol. 2026 Sep 30;64(9):e2608018. doi: 10.71150/jm.2608018 (PMC13688130; doi:10.71150/jm.2608018)
Supplement: Supplementary file 2 [file jm-2608018-Supplementary-Fig-S2.pdf]

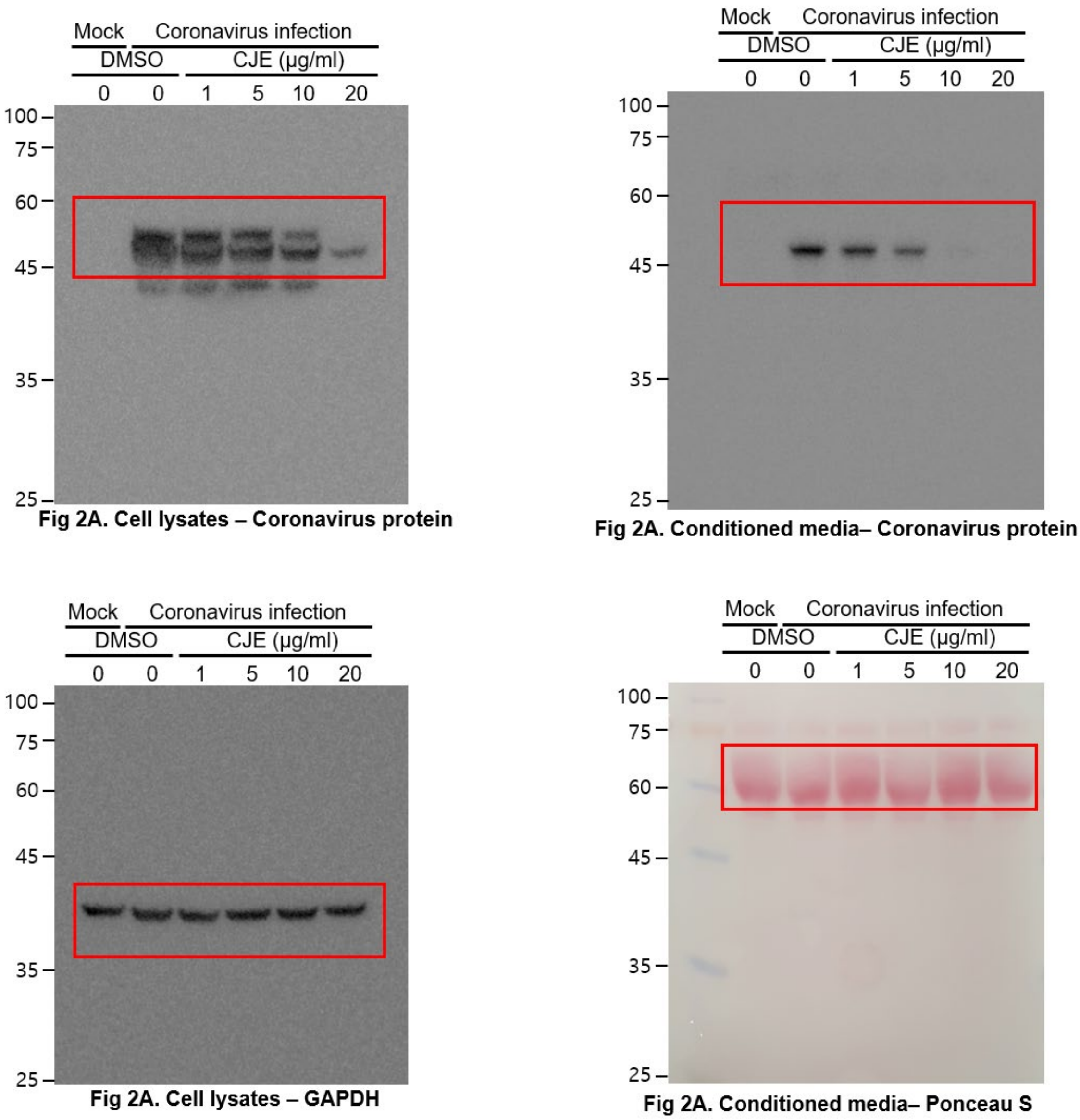

Fig. S2. Uncropped original Western blot images for Fig. 2A.
