# Supplementary material for: Antiviral activity of Cirsium japonicum var. spinossimum extract and its flavonoid cirsimaritin against human coronavirus OC43
Source: J Microbiol. 2026 Sep 30;64(9):e2608018. doi: 10.71150/jm.2608018 (PMC13688130; doi:10.71150/jm.2608018)
Supplement: Supplementary file 4 [file jm-2608018-Supplementary-Fig-S4.pdf]

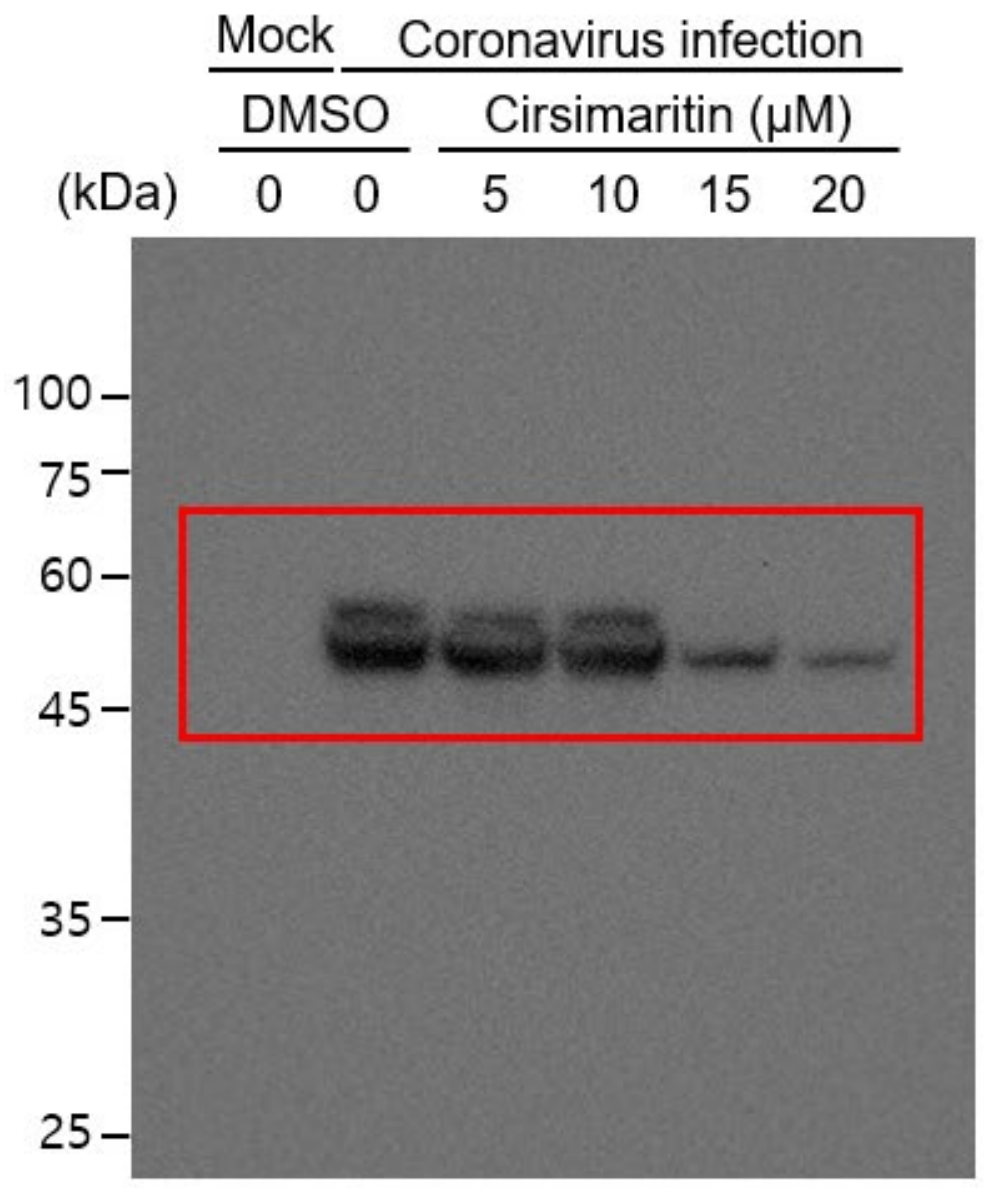

Fig 5D. Cell lysates – Coronavirus protein

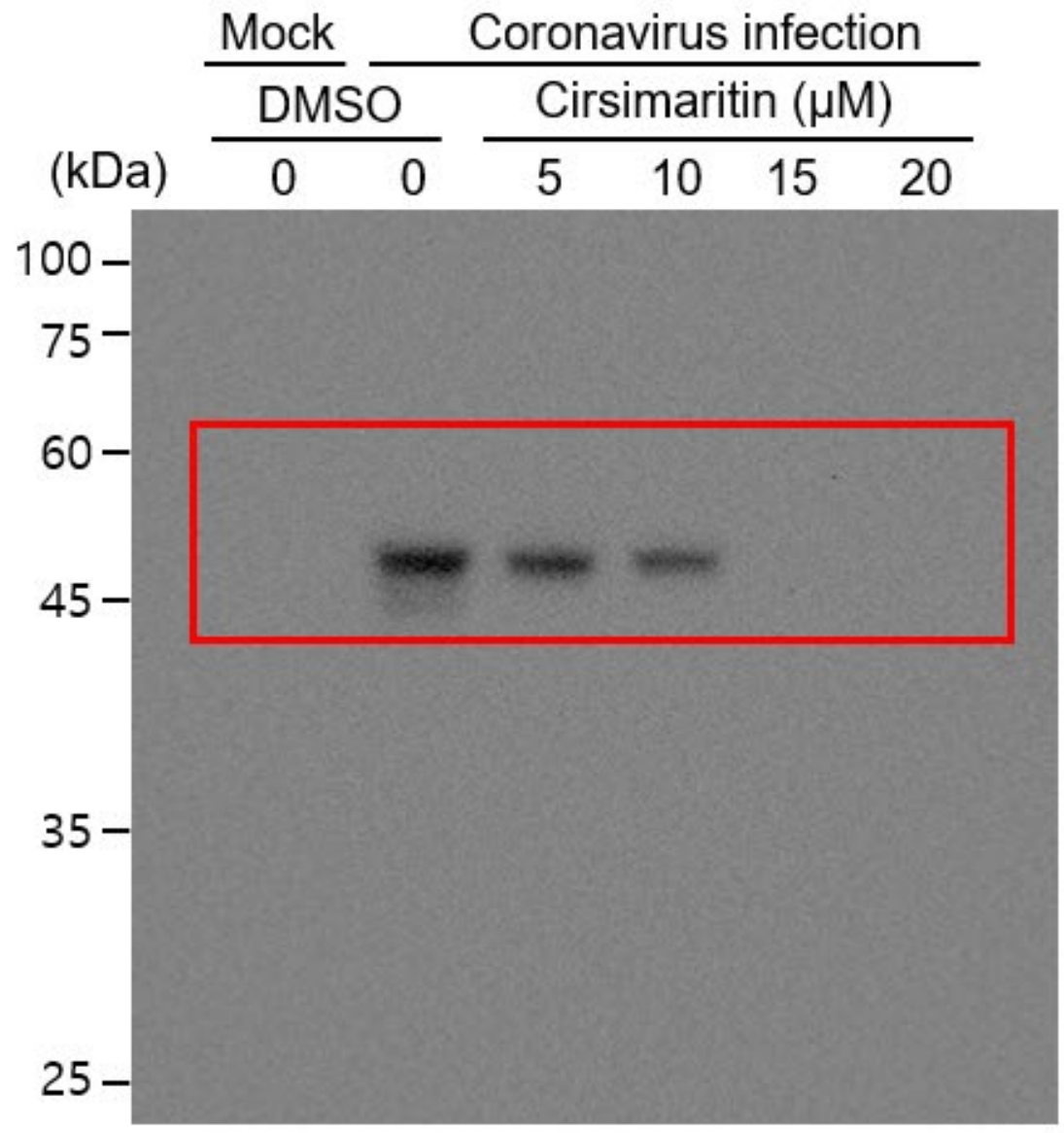

Fig 5D. Conditioned media– Coronavirus protein

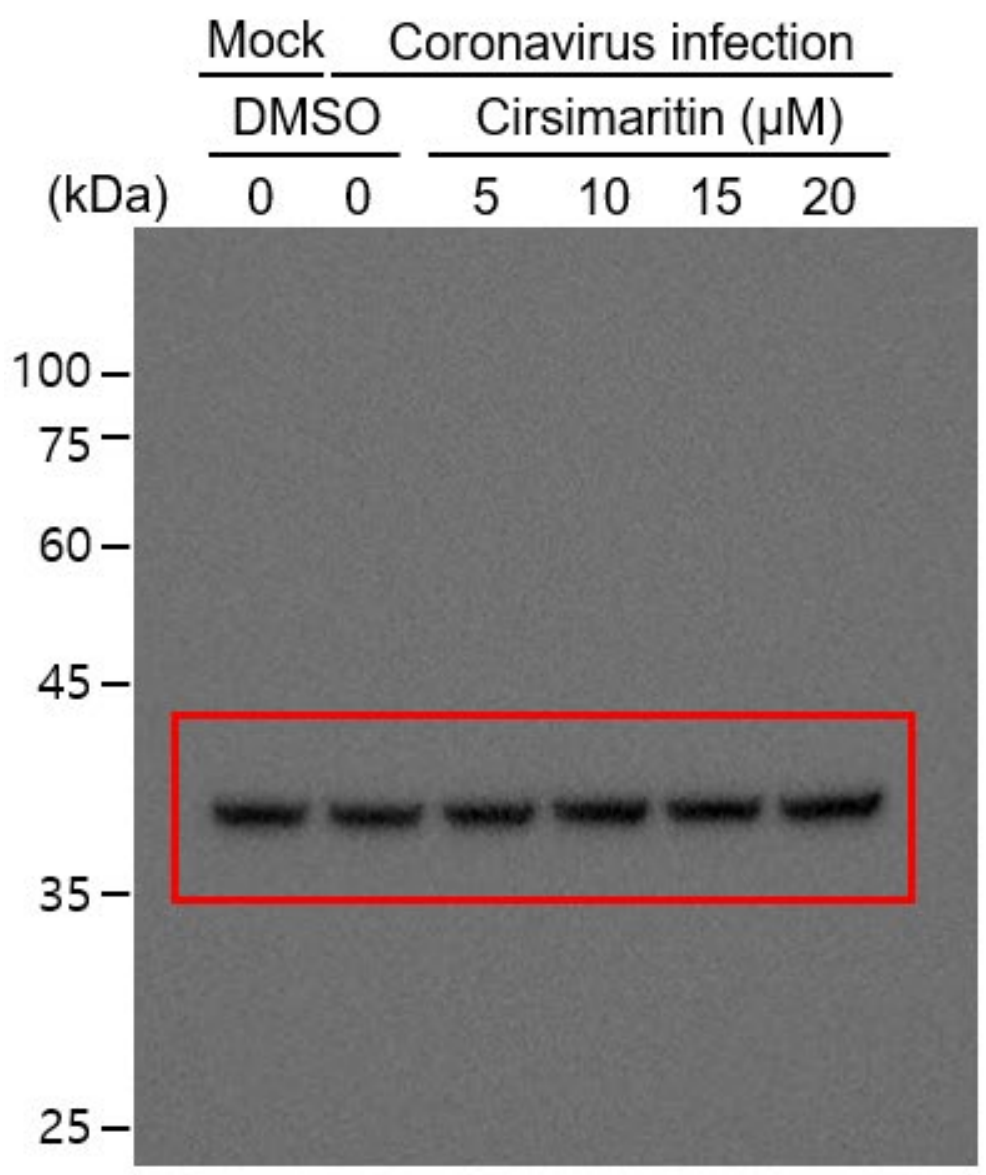

Fig 5D. Cell lysates – GAPDH

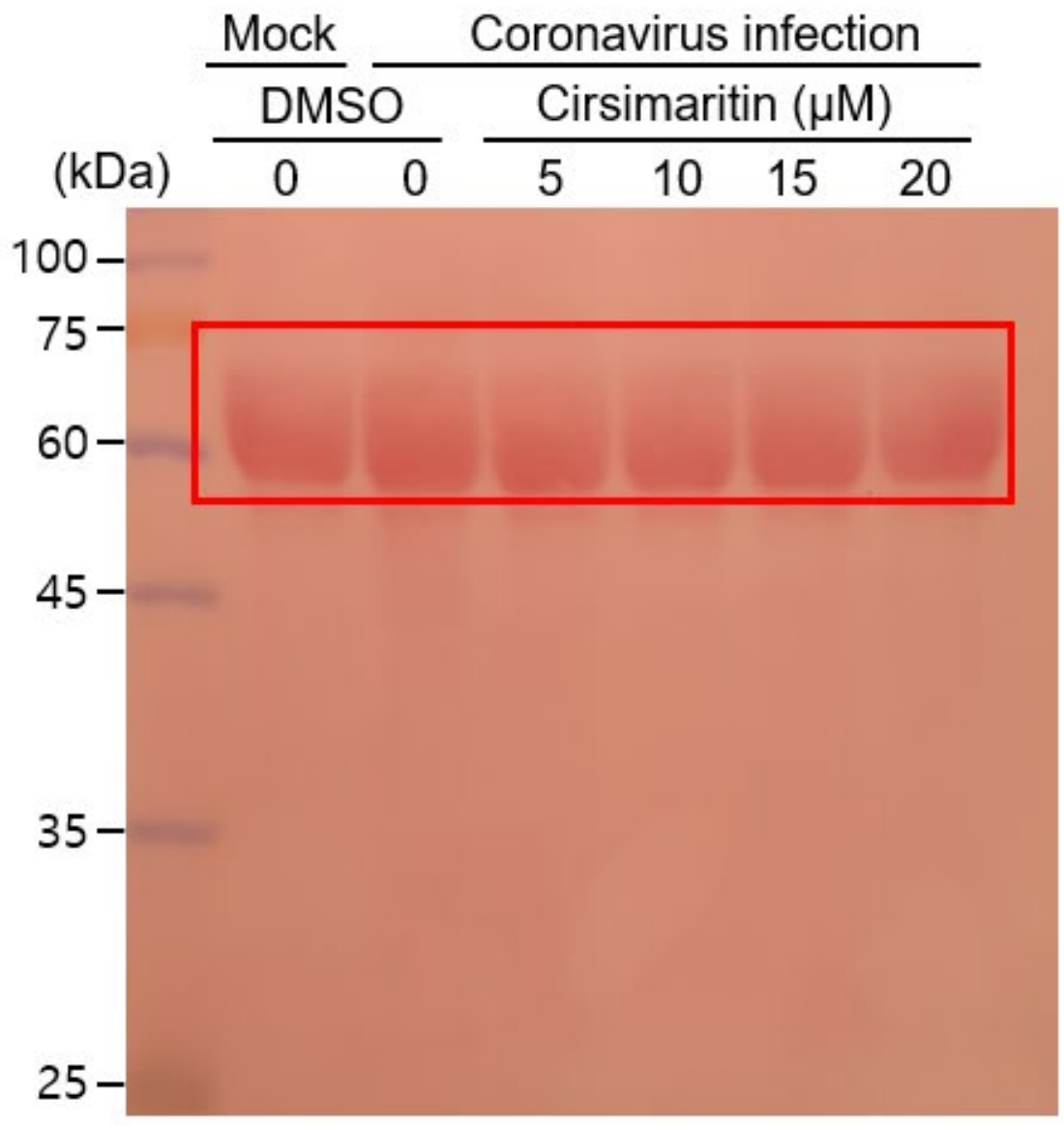

Fig 5D. Conditioned media– Ponceau S

Fig. S4. Uncropped original Western blot images for Fig. 5D.
